# Diffusion tensor imaging changes in patients with glioma-associated seizures

**DOI:** 10.1007/s11060-022-04139-9

**Published:** 2022-11-07

**Authors:** Marius Marc-Daniel Mader, Daniel Deuter, Thomas Sauvigny, Patrick Borchert, Tobias D. Faizy, Maxim Bester, Manfred Westphal, Katharina Rosengarth, Nils O. Schmidt, Jan Sedlacik, Lasse Dührsen

**Affiliations:** 1 Department of Neurosurgery, University Medical Center Hamburg-Eppendorf, Martinistraße 52, 20246 Hamburg, Germany; 2 Diagnostic and Interventional Neuroradiology, University Medical Center Hamburg-Eppendorf, Martinistraße 52, 20246 Hamburg, Germany; 3 Institute for Stem Cell Biology and Regenerative Medicine, Stanford University School of Medicine, 265 Campus Drive, Stanford, CA 94305 USA; 4 Department of Neurosurgery, University Medical Center Regensburg, Franz-Josef-Strauss-Allee 11, 93053 Regensburg, Germany; 5 Department of Neuroimaging and Neurointervention, Stanford University School of Medicine, 300 Pasteur Dr, Stanford, CA 94305 USA; 6 Centre for the Developing Brain and Biomedical Engineering Department, School of Biomedical Engineering & Imaging Sciences, King’s College London, London, UK

**Keywords:** Glioma, Seizures, Diffusion Tensor Imaging, Humans, White Matter, Epilepsy

## Abstract

**Introduction:**

Structural white matter changes associated with certain epilepsy subtypes have been demonstrated using diffusion tensor imaging (DTI). This observational study aims to identify potential water diffusion abnormalities in glioma patients with associated seizures.

**Methods:**

Two cohorts from two centers were analyzed independently: (A) Prospectively recruited patients diagnosed with glioma who received preoperative DTI to measure mean diffusivity (MD) and fractional anisotropy (FA) in regions-of-interest (ROIs) including the marginal tumor zone (TU), adjacent peritumoral white matter as well as distant ipsilateral and contralateral white matter and cortex. Data were compared between patients with and without seizures and tested for statistical significance. (B) A retrospective cohort using an alternative technical approach sampling ROIs in contrast enhancement, necrosis, non-enhancing tumor, marginal non-enhancing tumor zone, peritumoral tissue, edema and non-tumorous tissue.

**Results:**

(A) The prospective study cohort consisted of 23 patients with 12 (52.2%) presenting with a history of seizures. There were no significant seizure-associated differences in MD or FA for non-tumor white matter or cortical areas. MD-TU was significantly lower in patients with seizures (p = 0.005). (B) In the retrospective cohort consisting of 46 patients with a seizure incidence of 50.0%, significantly decreased normalized values of MD were observed for non-enhancing tumor regions of non-glioblastoma multiforme (GBM) cases in patients with seizures (p = 0.022).

**Conclusion:**

DTI analyses in glioma patients demonstrated seizure-associated diffusion restrictions in certain tumor-related areas. No other structural abnormalities in adjacent or distant white matter or cortical regions were detected.

**Supplementary Information:**

The online version contains supplementary material available at 10.1007/s11060-022-04139-9.

## Introduction

Seizures are a common symptom associated with glioma with an incidence that ranges between 30 and 62% in patients with glioblastoma multiforme (GBM) and 65–85% with low-grade glioma [1]. Even though several predictive markers have been reported including demographic, clinical and histopathological features [2–11], the underlying pathophysiological mechanisms are not completely understood yet [12]. Magnetic resonance imaging (MRI) with diffusion tensor imaging (DTI) could potentially provide additional information about these mechanisms by demonstrating potential water diffusion abnormalities.

DTI has previously been identified as a tool to examine structural white matter changes associated with certain epilepsy subtypes [13–18]. A preceding study revealed reduced anisotropy and increased diffusivity in seizure patients with non-progressive acquired lesions such as after head injury or infarction [15]. Evidence for disrupted white matter architecture was also found in temporal lobe epilepsy with an increased mean diffusivity (MD) in the hippocampus and its primary and remote connections, as well as reduced fractional anisotropy (FA) [13, 16, 17]. Particularly FA reduction seems to be also associated with refractoriness [14]. Moreover, there is evidence that structural abnormalities fail to normalize despite seizure freedom after surgical treatment [18].

Despite these intriguing findings, DTI has not been used to study tumor associated seizures yet. This study therefore aims to explore changes of MD and FA derived from DTI in glioma patients with seizures.

## Methods

Two cohorts from two tertiary medical centers were analyzed independently. This study was approved by the local ethics committees (Ethik-Kommission der Ärztekammer Hamburg, PV3636; Ethikkomission der Universität Regensburg, 21-2549-104) and was performed in accordance with international ethical standards and institutional guidelines/regulations. Informed written consent was obtained from all study participants.

### Cohort A

Patients diagnosed with a supratentorial intra-axial lesion were prospectively recruited for a primary explorative cohort, referred to as cohort A. Preoperatively, an MRI was performed with a 3 Tesla Skyra MAGNETOM (Siemens Healthineers, Erlangen, Germany) and 32 receive channel head coil. DTI scanning parameters were TE = 73 ms, TR = 4600 ms, b = 1000 s/m^2^, Multi Directional Diffusion Weighting factor = 64, image matrix = 128 × 128, in-plane resolution = 1.9 × 1.9 mm^2^, 34 slices with 4 mm slice thickness and no gap, parallel acquisition acceleration factor = 2 and partial Fourier factor = 6/8, dynamic field correction = on, readout bandwidth = 1628 Hz/pixel.

Patients included in data analysis had a confirmed histological diagnosis of either glioblastoma, IDH-wildtype (CNS WHO grade 4), astrocytoma, IDH-mutant (CNS WHO grade 2–4), or oligodendroglioma, IDH-mutant and 1p/19q-codeleted (CNS WHO grade 2–3). Exclusion criteria were unclear history of seizures and poor visibility of tumor in DTI imaging, particularly in trace, which would have led to inaccurate ROI specification.

**Fig. 1:**
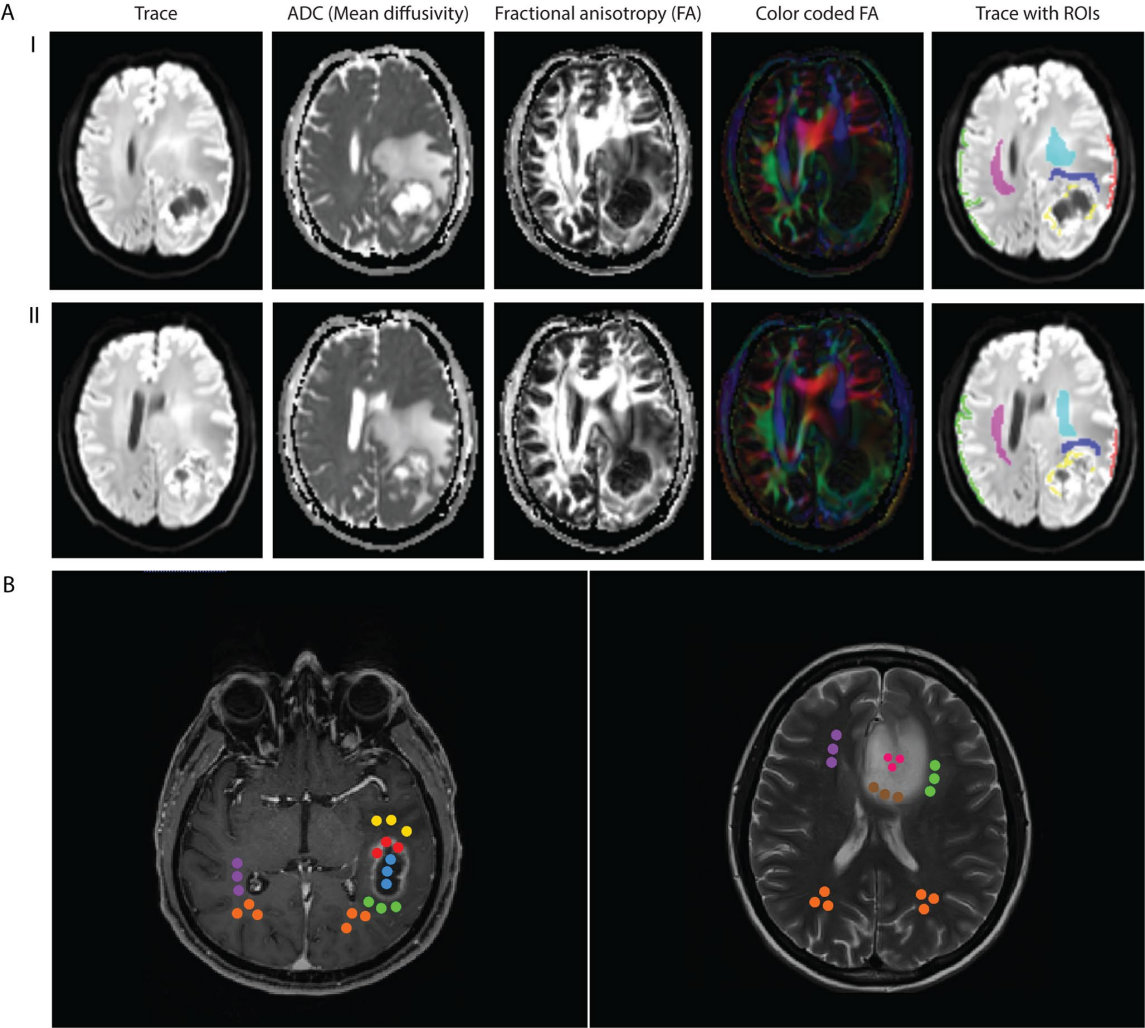
(A) Representative images of a DTI scan are presented for two different axial slices from the same patient (I & II). The regions of interest (ROI) for the analysis of our first cohort A are color labeled and consist of the marginal tumor zone (yellow), white matter adjacent to the tumor (dark blue), ipsilateral (red) and contralateral (green) cortex as well as ipsilateral (cyan) and contralateral (magenta) white matter. (B) Representative slices of a T1-MPRAGE image of a patient with GBM, IDH-wildtype (left), and a T2-weighted image of a patient with astrocytoma, IDH-mutant, CNS WHO Grade 2 (right) are shown. Colored circles illustrate the definition of regions of interest (ROI) for our second cohort B. In GBM patients and IDH-mutant astrocytoma CNS WHO Grade 4, three spheric ROIs were defined in each contrast enhancement (red), necrosis (blue), peritumoral white matter (green), edema (yellow), the area contralateral to the tumor in the opposite hemisphere (violet) as well as ipsi- and contralateral controls frontal or occipital depending on tumor position (orange, left image). In all other tumor entities (right image), three spheric ROIs were defined in each non-enhancing tumor (pink), marginal non-enhancing tumor zone (brown), peritumoral white matter (green), edema (not present), the area contralateral to the tumor in the opposite hemisphere (violet) as well as ipsi- and contralateral controls frontal or occipital depending on tumor position (orange) and contrast enhancement/ necrosis if present

Postprocessed MD and FA maps were exported from the SIEMENS console and analyzed with MRIcron (Version: 2MAY2016; https://www.nitrc.org/projects/mricron). ROIs were defined as tumor tissue of the marginal zone of the lesion (TU), white matter adjacent to the lesion (AWM), as well as distant ipsilateral and contralateral grey and white matter as shown in Fig. 1 A. The marginal tumor zone was chosen over the whole tumor in order to sample live tissue and avoid cystic or necrotic areas. ROIs were manually drawn over multiple slices in which tumor structures could be clearly identified. ROIs were similar for all glioma entities. Mean values of FA and MD were exported for each ROI. Additionally, perifocal edema volume in the vicinity of the tumor mass was determined by using a non-threshold-based manual segmentation tool (Horos, Horosproject.org).

Collection of clinical data included demographics, history of seizures, tumor location and histopathological specifications. These included WHO grading based on the 2021 WHO classification of tumors of the central nervous system (5th revised edition; older diagnoses based on the 2016 WHO classification were transformed to the new 2021 classification), immunohistochemistry for mutant R132H IDH1 protein for all samples, and 1p/19q codeletion status for samples with oligodendroglial histological morphology. For this study, if 1p/19q was not tested, it was assumed as wildtype. The methylation status of the promotor for the gene of O-6-methylguanine-DNA methyltransferase (MGMT) was assessed by methylation-specific PCR. Differences between subgroups consisting of patients with and without seizures were tested for statistical significance.

Statistical analyses were performed using SPSS (Version 24.0, IBM Inc., Armonk, New York, USA). Data are presented as mean ± standard deviation (SD) for continuous variables, and as numbers and percentages for categorical variables. Categorical variables were tested for significant differences using either Pearson Chi-Square or Fisher’s exact test, dependent on sample size. Statistical differences between independent continuous variables were tested either with the t-test or Mann-Whitney U test, dependent on normality distribution of samples. A p-value of < 0.05 was considered statistically significant.

### Cohort B

A second center cohort of patients with intra-axial lesions and preoperative DTI was retrospectively analyzed, referred to as cohort B. Patients with unclear history of seizures were excluded. We included patients with confirmed histological diagnosis of glioblastoma, IDH-wildtype (CNS WHO grade 4), gliosarcoma (CNS WHO grade 4), astrocytoma, IDH-mutant (CNS WHO grade 2–4), oligodendroglioma, IDH-mutant and 1p/19q-codeleted (CNS WHO grade 2–3), ganglioglioma (CNS WHO grade 1), and pilocytic astrocytoma (CNS WHO grade 1). Additionally, three tumors formerly classified as anaplastic astrocytoma/ oligodendroglioma WHO °III, IDH-wildtype, NOS, were part of the study.

Preoperative DTI imaging was performed at a 3 Tesla Skyra MAGNETOM (Siemens Healthineers, Erlangen, Germany) with 30 gradient directions. DTI scanning parameters were TE = 95ms, TR = 5200ms, FoV = 1610 × 1610, flip angle = 90°, image matrix 256 × 256, voxel size = 0.89 × 0.89 × 3 mm.

To adjust for methodological aspects, a different approach was chosen for DTI analyses than in the explorative cohort A using FSL 6.0.3 (fsl.fmrib.ox.ac.uk/, [19–21]) to avoid method-associated artifacts. DICOMs were converted to NIFTI format using the dcm2-nii tool (people.cas.sc.edu/rorden/mricron/). Data were corrected for proband movement errors and eddy current induced errors using EDDY [22]. Diffusion tensor models were fitted to individual voxels using DTIFIT leading to patient-specific FA and MD maps. After skull stripping of MR images (MPR, T2, FLAIR) using BET [23], co-registration of the different MRI sequences was performed with FLIRT (T2 to MPR, FLAIR to MPR, [24–26]) and the epi_reg script included in FLIRT (DTI to MPR). Results were controlled visually with respect to anatomic accuracy in direct semi-transparent overlay.

ROIs were defined based on MPR-/ T2- and FLAIR sequences depending on the visibility of the particular tumor entity in the specific sequences. To avoid possible effects due to different ROI volumes, three-dimensional spheres with a fixed diameter of 4 mm were defined in subcortical white matter. In this cohort, two sets of ROIs were used depending on glioma entity. In GBMs and IDH-mutant astrocytoma CNS WHO Grade 4, we defined three spheres in each contrast enhancement, as well as spheres in areas of necrosis, peritumoral white matter (AWM), edema, the area contralateral to the tumor in the opposite hemisphere as well as ipsi- and contralateral controls frontal or occipital depending on tumor position. In the other tumor entities, ROIs were defined in non-enhancing tumor (NENH), marginal non-enhancing tumor zone (MARG-NENH), contrast enhancement if present, necrosis if present as well as the above-mentioned control regions. The TU ROI from cohort A is therefore represented by both the contrast enhancement ROI and MARG-NENH ROI in cohort B. Figure 1B illustrates the definition of ROIs. Minimum, mean and maximum FA and MD were exported for each ROI.

Additionally, quotients normalizing contrast enhancement, necrosis, edema, non-enhancing tumor areas and the marginal non-enhancing tumor zone to the adjacent peritumoral white matter, as well as the area “opposite” to the tumor and ipsi- and contralateral controls were calculated. Collection of clinical data and statistical analyses using SPSS 25 (IBM Inc., Armonk, New York, USA) were performed as mentioned above.

## Results

### Cohort A

Twenty-nine patients have been included in the explorative cohort A and received preoperative DTI imaging. Of these, six patients were excluded - one due to diagnosis of an astroblastoma, three due to unclear history of seizures, and two due to unusable imaging data. The final study cohort used for data analysis consisted of 23 patients. The tumor was a new diagnosis in 22 patients (95.7%) and a recurrent GBM in one patient. Twelve patients reported a history of seizure, which is further detailed in Table 1. Patients with seizures were on average significantly younger (47.1 ± 8.4 vs. 57.1 ± 13.2 years). Most common pathology was GBM in 47.8% of cases. About half of the study cohort was IDH1 mutated (52.2%) and seven samples had a confirmed 1p/19q codeletion (30.4%). Patients without a history of seizures suffered mostly from GBM (63.6%). Further clinical details of cohort A are shown in Table 2.

**Table 1:** Clinical presentation of glioma associated seizures from patients of the exploratory cohort A

		n	Percent
Number of seizures	one time	7	58.3
	multiple	4	33.3
	unclear	1	8.3
Presentation	generalized	6	50.0
	focal	4	33.3
	mixed	1	8.3
	unclear	1	8.3
Time from first seizure to MRI	< 7 days	4	33.3
	< 1 month	4	33.3
	> 1 month	4	33.3

**Table 2:** Demographic and histopathologic characteristics of the exploratory cohort A

		All patients (n = 23)	Seizure patients (n = 12)	Seizure-free patients (n = 11)	p value (seizure vs. non-seizure)
Age	years	51.9 ± 11.9	47.1 ± 8.4	57.1 ± 13.2	0.040
Sex	female	3 (13.0%)	2 (16.7%)	1 (9.1%)	1.000
Diagnosis	Glioblastoma, IDH-wildtype	11 (47.8%)	4 (33.3%)	7 (63.6%)	0.103
	Astrocytoma, IDH-mutant	5 (21.7%)	2 (16.7%)	3 (27.3%)	
	Oligodendroglioma, IDH-mutant & 1p/19q-codeleted	7 (30.4%)	6 (50.0%)	1 (9.1%)	
CNS WHO grade	4	12 (52.2%)	4 (33.3%)	8 (72.7%)	0.125
	3	6 (26.1%)	5 (41.7%)	1 (9.1%)	
	2	5 (21.7%)	3 (25.0%)	2 (18.2)	
IDH-1 status	mutated	12 (52.2%)	8 (66.7%)	4 (36.4%)	0.220
1p19q status	codeleted	7 (30.4%)	6 (50.0%)	1 (9.1%)	0.069
	wildtype or missing	16 (69.6%)	6 (50.0%)	10 (90.9%)	
MGMT status	methylated	17 (73.9%)	9 (75.0%)	8 (72.7%)	1.000
	unmethylated or missing	4 (17.4%)	2 (16.7%)	2 (18.2%)	
	N/A	2 (8.7%)	1 (8.3%)	1 (9.1%)	
Localization	frontal	9 (39.1%)	5 (41.7%)	4 (36.4%)	0.750
	parietal	6 (26.1%)	3 (25.0%)	3 (27.3%)	
	temporal	7 (30.4%)	3 (25.0%)	4 (36.4%)	
	occipital	1 (4.3%)	1 (8.3%)	0 (0.0%)	
Edema volume	cm³	41.6 ± 44.8	33.0 ± 35.6	51.0 ± 53.2	0.268

**Fig. 2:**
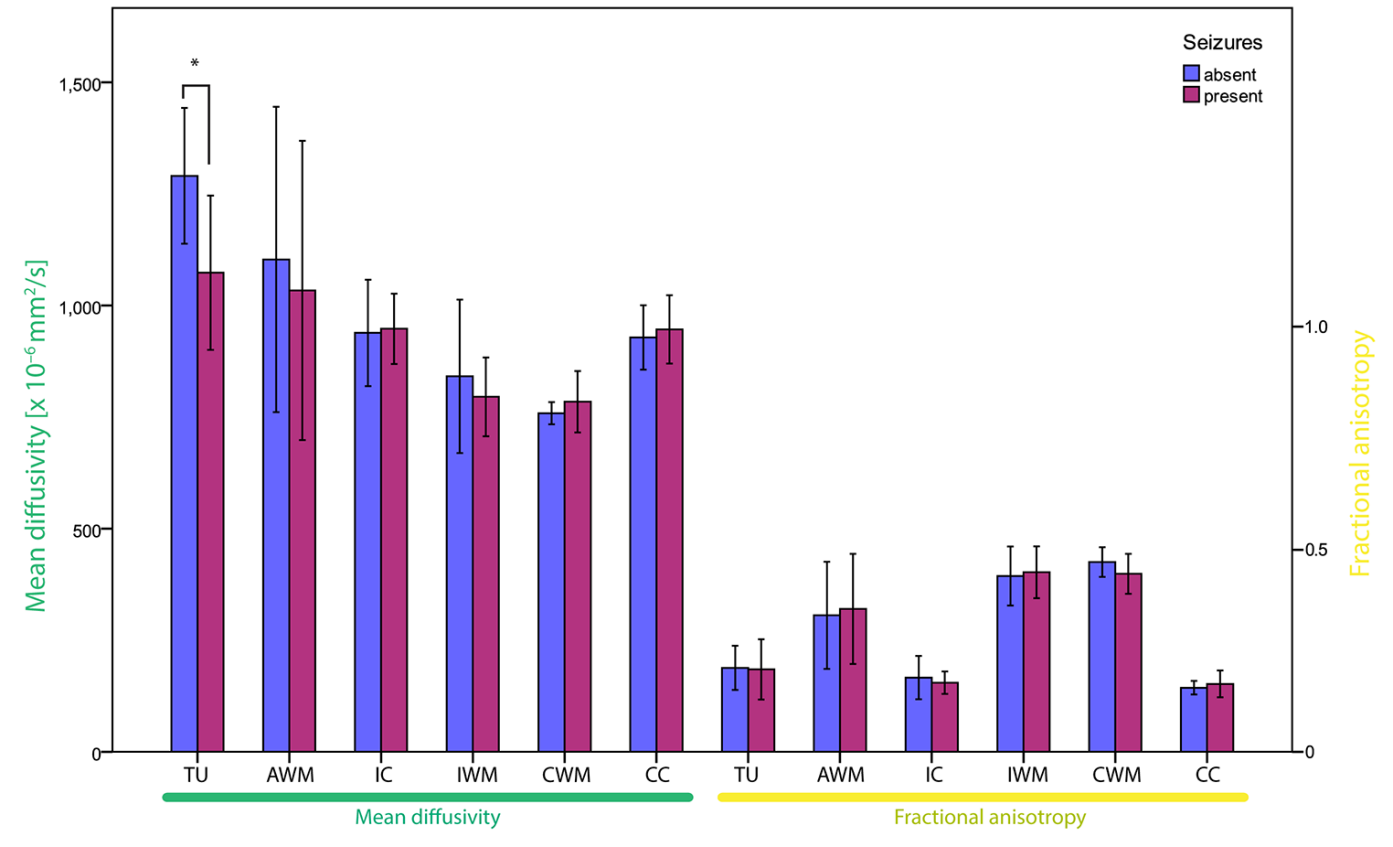
The bar chart demonstrates the mean diffusivity and fractional anisotropy (mean ± SD) for different regions of interest for the exploratory cohort A. These were the marginal zone of the tumor (TU), white matter adjacent to the tumor (AWM), ipsilateral cortex (IC) and white matter (IWM) as well as contralateral white matter (CWM) and cortex (CC). Data was dichotomized into patients with and without history of seizures. Differences between these two groups were tested for statistical significance (*) in different regions of interest

MD appeared lowest in distant white matter, higher in distant cortical areas, and highest in association to the tumor lesion as shown in Fig. 2. The TU area (1177.2 ± 194.2 × 10^− 6^ mm^2^/s) and AWM (1067.0 ± 332.6 × 10^− 6^ mm^2^/s) showed both significantly elevated MD compared to ipsilateral distant white matter (817.3 ± 133.8 × 10^− 6^ mm^2^/s, p < 0.001). However, only MD-TU was significantly higher than ipsilateral cortex (943.3 ± 97.9 × 10^− 6^ mm^2^/s, p < 0.001). Distant cortical areas demonstrated the lowest and distant white matter areas the highest FA. FA of TU was significantly lower compared to ipsilateral distant white matter (0.1863 ± 0.0583 vs. 0.3983 ± 0.0607, p < 0.001), but showed no significant difference to the ipsilateral cortex (0.1603 ± 0.0379). AWM was significantly different from both ipsilateral distant white matter and cortex (0.3132 ± 0.1193, p < 0.001).

**Fig. 3:**
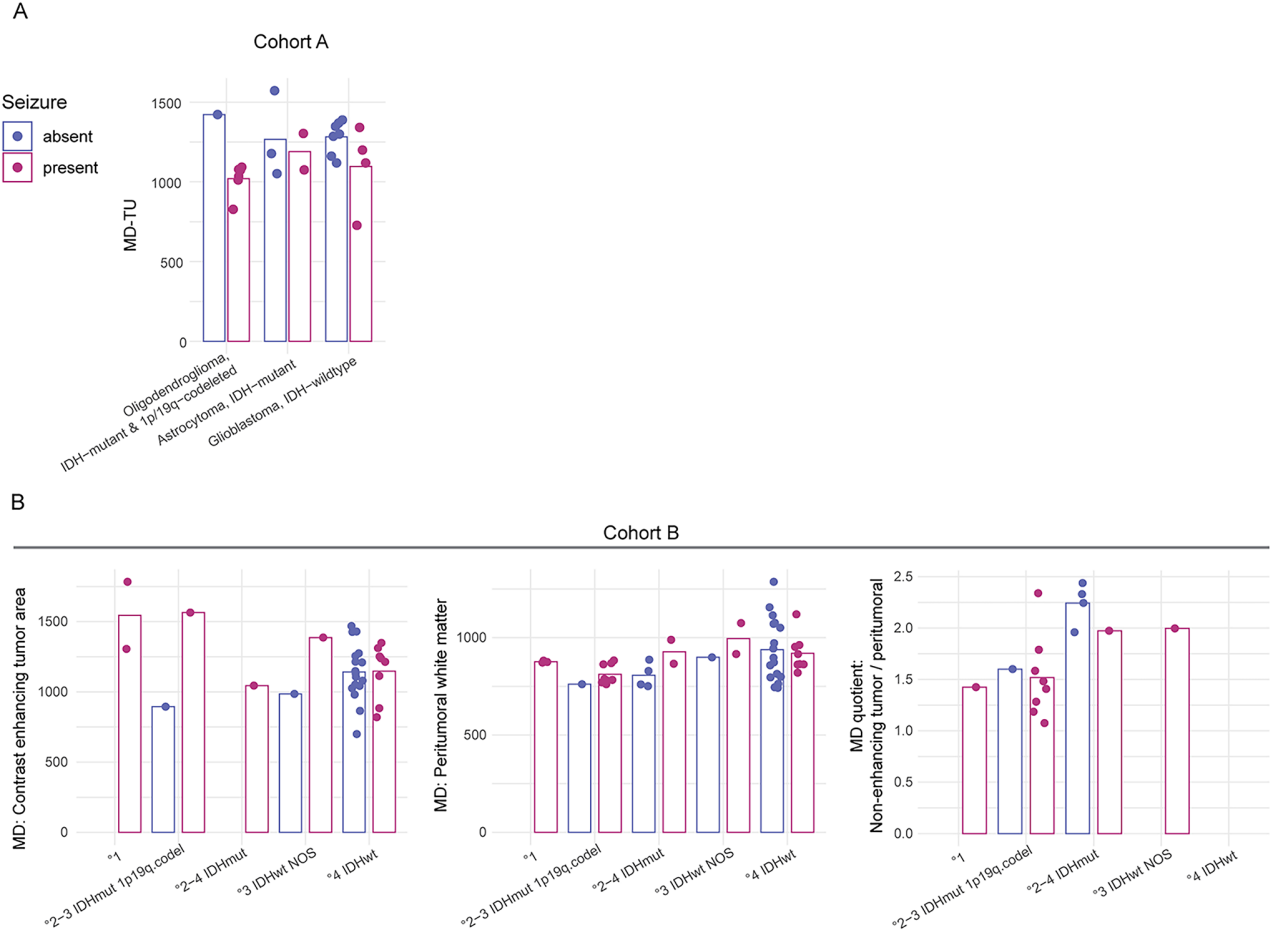
Bar charts illustrate the distribution of mean diffusivity (MD) of key regions of interest in cohort A (A) and cohort B (B) between different glioma entities separated for seizure status. Bars represent the mean; absolute units are 10^− 6^ mm^2^/s

When analyzing differences in DTI between patients with and without previous seizures there were no statistically significant differences in MD or FA of distant or adjacent white matter or cortical areas. However, MD-TU was significantly lower in patients with seizures (1073.6 ± 172.8 vs. 1290.3 ± 152.3 × 10^− 6^ mm^2^/s, p = 0.005), whereas no significant difference in FA was present. We found no correlation between MD-TU and time interval from first seizure to MRI (Pearson correlation coefficient 0.039, p = 0.904). The distribution of MD-TU between the major glioma entities represented in cohort A is demonstrated in Fig. 3 A.

### Cohort B

Cohort B consisted of 46 patients after exclusion of 6 subjects with unclear history of seizure. Four patients with recurrent tumor or malignant progression of previous gliomas were part of the cohort. No significant differences could be found regarding age and sex when comparing patients with seizures (23 patients, 50.4 ± 20.2 years) and patients without seizures (23 patients, 57.4 ± 14.8 years). More than half of the cohort (52.2%) suffered from GBM. The majority of GBM patients had no seizures (70.8%). The 1p/19q status significantly differed between the two groups (43.5% in the seizure group vs. 4.3% in the non-seizure group, p = 0.005). Detailed information about the composition of cohort B regarding tumor characteristics can be found in Table 3.

**Table 3:** Demographic and histopathologic characteristics of cohort B

		All patients (n = 46)	Seizure patients (n = 23)	Seizure-free patients (n = 23)	p value (seizure vs. non-seizure)
Age	Years	53.91 +/- 17.85	50.43 +/- 20.22	57.39 +/- 14.75	0.222
Sex	Female	20 (43.5%)	12 (52.2%)	8 (34.8%)	0.234
Diagnosis	Glioblastoma, IDH-wildtype	24 (52.2%)	7 (30.4%)	17 (73.9%)	**0.005**
	Gliosarcoma, IDH-wildtype	1 (2.2%)	1 (4.3%)	0 (0.0%)	
	Astrocytoma, IDH-mutant	6 (13.0%)	2 (8.7%)	4 (17.4%)	
	Oligodendroglioma, IDH-mutant & 1p/19q-codeleted	9 (19.6%)	8 (34.8%)	1 (4.3%)	
	Formerly diagnosed as anaplastic Astrocytoma/ Oligodendroglioma WHO °III, IDH-wt, NOS	3 (6.5%)	2 (8.7%)	1 (4.3%)	
	Ganglioglioma	1 (2.2%)	1 (4.3%)	0 (0.0%)	
	Pilocytic astrocytoma	2 (4.3%)	2 (8.7%)	0 (0.0%)	
CNS WHO grade	4	26 (56.5%)	9 (39.1%)	17 (73.9%)	0.051
	3	13 (28.3%)	9 (39.1%)	4 (17.4%)	
	2	4 (8.7%)	2 (8.7%)	2 (8.7%)	
	1	3 (6.5%)	3 (13.0%)	0 (0.0%)	
IDH-1 status	mutated	15 (32.6%)	10 (43.5%)	5 (21.7%)	0.070
1p19q status	Codeleted	11 (23.9%)	10 (43.5%)	1 (4.3%)	**0.005**
	Wildtype or missing	35 (76.1%)	13 (56.5%)	22 (95.7%)	
MGMT status	Methylated	30 (65.2%)	16 (69.6%)	14 (60.9%)	0.241
	Unmethylated or missing	12 (26.1%)	4 (17.4%)	8 (34.8%)	
	N/A	4 (8.7%)	3 (13.0%)	1 (4.3%)	
Localization	Frontal	23 (50.0%)	14 (60.9%)	9 (39.1%)	0.418
	Parietal	10 (21.7%)	3 (13.0%)	7 (30.4%)	
	Temporal	10 (21.7%)	5 (21.7%)	5 (21.7%)	
	occipital	1 (2.2%)	0 (0.0%)	1 (4.3%)	
	multifocal	2 (4.3%)	1 (4.3%)	1 (4.3%)	

Overall for cohort B, MD was slightly elevated in peritumoral white matter, necrosis, edema, non-enhancing parts of the tumor in non-GBM cases, the non-enhancing marginal zone in non-GBMs, and in contrast enhancement compared to control ROIs (Supplementary Fig. 1). Patients with seizures showed a non-significant trend of lower MD values in non-enhancing tumor areas (1329.5 ± 336.3 vs. 1693.8 ± 341.6 × 10^− 6^ mm^2^/s) and in the marginal zone of the non-enhancing area (1246.5 ± 307.8 vs. 1488.7 ± 329.1 × 10^− 6^ mm^2^/s). In contrast enhancement, MD values showed an opposite distribution with higher MD values in the seizure group (1251.6 ± 258.3 vs. 1120.8 ± 200.4 × 10^− 6^ mm^2^/s).

In regards to FA, lower values of FA were found in contrast enhancement, necrosis, edema, peritumoral white matter, non-enhancing tumor parts in non-GBM cases and in the marginal zone of non-enhancing tumor areas in non-GBMs compared to control ROIs (Supplementary Fig. 2). No significant differences between FA values of ROIs of the seizure and non-seizure group were detected.

MD and FA values within the ROIs of contrast enhancement, necrosis, edema, non-enhancing tumor in non-GBM cases and the marginal non-enhancing tumor zone in non-GBMs were normalized to the values within the ROIs of adjacent peritumoral white matter (AWM), the area “opposite” to the tumor and ipsi-/ contralateral controls. In relation to MD, significant results could be found for the ROI of non-enhancing tumor areas (NENH) normalized to the peritumoral white matter (seizures 1.5946 ± 0.3882 vs. no-seizures 2.1145 ± 0.3378, p = 0.022), to ipsilateral controls (1.6284 ± 0.4046 vs. 2.1320 ± 0.4261, p = 0.039) and to contralateral controls (1.6228 ± 0.4305 vs. 2.2032 ± 0.3748, p = 0.021) with significantly lower values in the seizure group. For other normalized quotients as well as FA, no significant differences were found. Normalized MD of non-enhancing tumor areas was higher in astrocytomas, IDH-mutant, compared to oligodendrogliomas, IDH-mutant and 1p/19q-codeleted (Fig. 3B).

## Discussion

In this study we describe diffusion properties of different brain and tumor regions in glioma patients using two different methodical approaches with the aim to explore potential seizure-specific changes. No structural abnormalities in adjacent or distant white matter, or cortical regions were detected. In the exploratory cohort A, it was found that tissue of the marginal tumor zone appeared more diffusion restricted in glioma patients suffering from seizures. A change in MD was also demonstrated in the second cohort B for non-enhancing tumor regions in non-GBM cases after normalization. Adversely, in GBM patients and IDH-mutant astrocytoma CNS WHO Grade 4 the contrast-enhancing part of the tumor showed an opposite behavior with higher MD values in the seizure group. A generally increased MD in the TU ROI compared to distant white matter is in agreement with previous observations [27].

MD of tumor regions was the only DTI parameter investigated in this study that differed significantly between patients with and without epilepsy. Other factors have previously been identified as predictive for seizures in glioma patients but also varied between different studies [2–11]. A relatively consistently reported variable is the IDH mutation status [4, 6, 7, 9]. Generally, the clinical and tumorbiological importance of molecular markers in glioma over histopathological diagnosis and WHO grading has been demonstrated before [32, 33]. Even though we were not able to show a significant effect in our smaller cohort A, a trend towards a differential distribution for both molecular markers IDH1 and 1p/19q between patients with and without seizures can be debated, especially with respect to the significant differences regarding the 1p/19q status in cohort B. Therefore, diffusion changes detected in seizure patients in this study should also be interpreted in the context of the underlying tumor biology. As demonstrated for cohort B the glioma type impacts diffusion changes. An association between DTI alterations, seizure risk, and marker gene mutations, like 1p/19q and IDH1, is possible (Fig. 3B). However, it appears that additional factors might impact seizure associated diffusion changes given that alterations of MD were observed in a similar direction within different groups of glioma entities, as shown for MD-TU in Fig. 3 A and for normalized MD in non-enhancing areas in Fig. 3B.

Except for changes of MD in the marginal tumor zone of the exploratory cohort A and normalized MD in the non-enhancing part of non-GBMs in cohort B, no other structural changes in the white matter were observed in our study, which is different from other types of epilepsy. Extensive temporal and extratemporal DTI abnormalities have been described for patients with temporal lobe epilepsy and mesial temporal sclerosis [16, 17]. An independent study group has shown increases in MD in areas of hippocampus, cingulum, fornix and the right external capsule in patients with temporal lobe epilepsy [13]. Moreover, an increase in MD and reduction in FA in the temporal white matter were found as markers for refractory mesial temporal lobe epilepsy [14]. The role and causality of these structural changes remains not definitively clarified. Based on DTI examinations in patients prior to and one year after epilepsy surgery for temporal lobe epilepsy, white matter DTI abnormalities appear irreversible despite absence of seizures, suggesting rather underlying structural abnormalities than functional changes [18]. However, in contrast to patients treated for long standing non-tumor associated epilepsy, patients with glioma associated seizures mostly present with a considerably shorter history of seizures. Absence of extra tumoral structural abnormalities in the current cohort may lead to the assumption that white matter changes shown in other studies might be rather the delayed consequence of chronic seizure activity than the cause. This might be vice versa for the glioma-associated alterations demonstrated here, particularly since there is no correlation with the time interval since first onset was observed.

Increased MD has previously been associated with cell loss in samples of hippocampus sclerosis, even allowing for the identification of subtypes depending on specific degrees of neuronal loss in certain hippocampal subfields and the respective MD values [28]. Similarly, lower MD values in meningiomas have been shown to be correlated with higher cell proliferation [29]. Increased MD in the context of glioma tissue in patients suffering from seizures might similarly be associated with lower cell densities. Another histopathological correlate could be changes of macromolecule content and architecture, e.g. of myelin. Also, accumulation of metabolites in the extracellular space may be a possible explanation considering the role of the glymphatic clearance pathway in other diseases [30]. In a rat model of pilocarpine-induced status epilepticus, reductions in MD were observed several weeks after the seizure along with a decrease in myelin staining [31]. However, previous considerations about proton physics in glioma tissue would contradict this with the hypothesis that elevated apparent diffusion coefficient levels might be related to the loss of proteins, such as myelin [27]. Whether decreased MD of tumor tissue in seizure patients is related to cellular alterations or swelling leading to a reduction of extracellular space with reduced diffusion of protons and / or to changes of the composition of macromolecules binding protons needs to be clarified in further studies. This would necessitate highly accurate mapping of collected specimen with the respective area in the MRI which we assessed insufficiently in the current study design with preoperative MRI. Usage of intraoperative MRI to clearly match tissue sampling with imaging could be advantageous to further explore the underlying histopathology of these structural abnormalities in glioma-associated seizure patients.

Methodically, two different approaches for region specific DTI analysis in glioma patients were used, both with innate advantages and limitations. In the exploratory cohort A, ROIs were defined manually only based on Trace images and accordingly independent from contrast enhancement. ROIs were therefore different in their respective volume. In cohort B, a more standardized sphere-based approach was used, and selection of ROIs was based on contrast-enhanced images. Accordingly, ROI volumes were clearly defined. A limitation regarding this approach is that the fixed volume of spheres may lead to slightly unspecific ROI representations in the case of small areas of interest. For example, contrast enhancements thinner than 4 mm were also represented by 4 mm measuring spheres sometimes leading to an involvement of peritumor tissue and necrotic tissue. Nevertheless, the design of this study with the use of two different methodologies in two independently analyzed cohorts was chosen to increase the methodological validity of findings, however, resulting in smaller patient numbers per cohort and less comparability between them.

In conclusion, DTI analyses in glioma patients demonstrated seizure-associated diffusion restrictions in certain tumor-related areas. However, no other structural abnormalities in adjacent or distant white matter or cortical regions were found. Further external studies are necessary to validate the pilot findings of this study. Potentially, DTI could serve as a tool to evaluate the likelihood of seizures in glioma patients and guide further diagnostic measures, like electroencephalogram and possible prophylactic actions. Future efforts to utilize DTI to determine the region of epileptogenesis within a neoplastic lesion might be supported by our data and could have important implications for surgical planning.

## Electronic supplementary material

Below is the link to the electronic supplementary material.

The bar chart demonstrates the mean diffusivity (mean ± SD, x 10^− 6^ mm^2^/s) for different regions of interest for cohort B. These were contrast enhancement (CE), necrosis (NEC), peritumoral white matter (AWM), edema (EDE), the area opposite to the tumor in the contralateral hemisphere (OPP), ipsi- and contralateral controls (COIPS/ COCON) as well as non-enhancing tumor parts (NENH) and marginal non-enhancing tumor tissue (MARG-NENH). Data was dichotomized into patients with and without history of seizures.

The bar chart demonstrates the fractional anisotropy (mean ± SD) for different regions of interest as above defined (see Fig. 5) for cohort B. Data was dichotomized into patients with and without history of seizures
