# Supplementary figures and images for: Diffusion tensor imaging changes in patients with glioma-associated seizures
Source: J Neurooncol. 2022 Nov 7;160(2):311–20. doi: 10.1007/s11060-022-04139-9 (PMC9722813; doi:10.1007/s11060-022-04139-9)

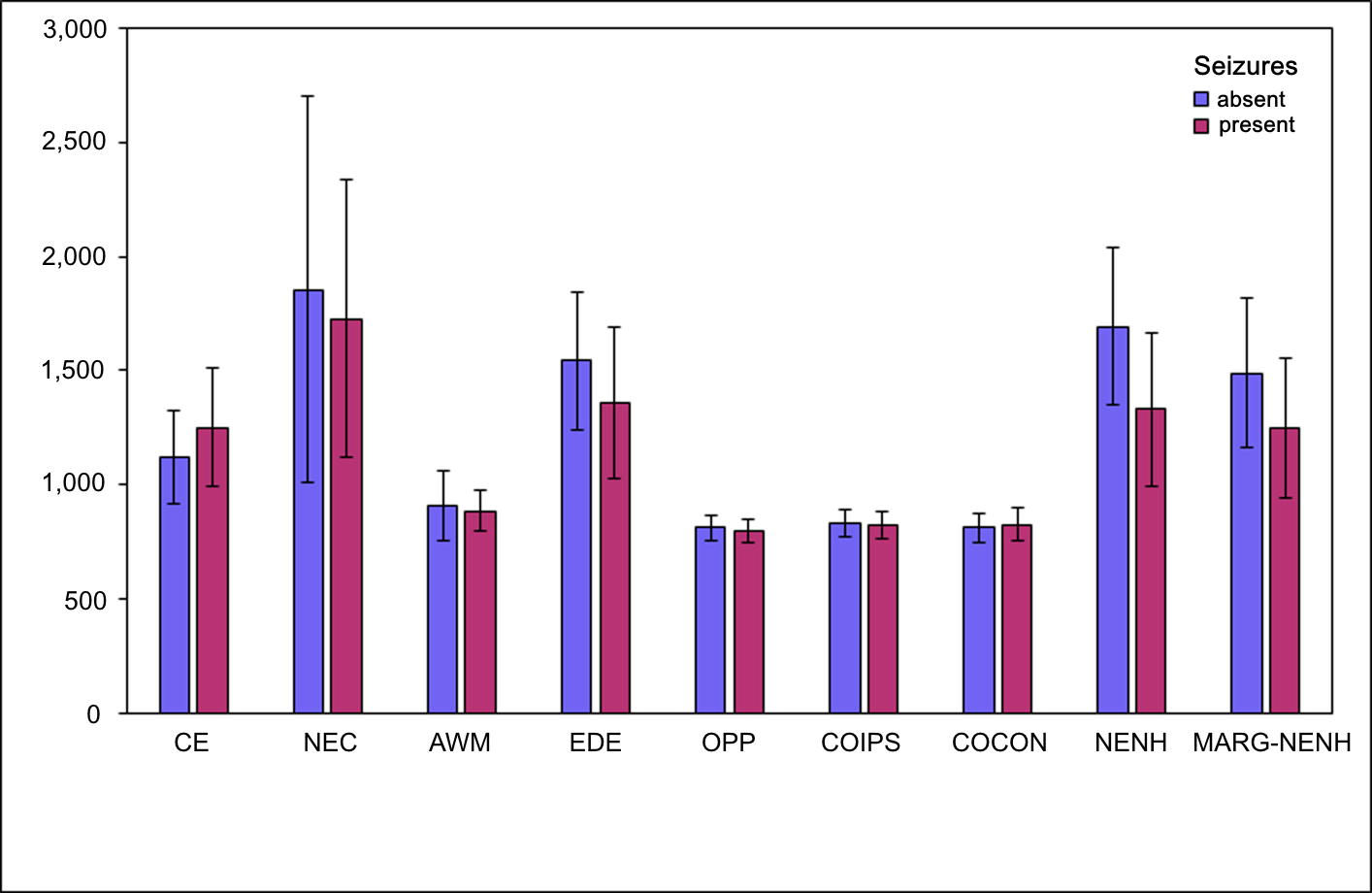

Supplement: Supplementary file 1 — The bar chart demonstrates the mean diffusivity (mean ± SD, x 10− 6 mm2/s) for different regions of interest for cohort B. These were contrast enhancement (CE), necrosis (NEC), peritumoral white matter (AWM), edema (EDE), the area opposite to the tumor in the contralateral hemisphere (OPP), ipsi- and contralateral controls (COIPS/ COCON) as well as non-enhancing tumor parts (NENH) and marginal non-enhancing tumor tissue (MARG-NENH). Data was dichotomized into patients with and without history of seizures. [file 11060_2022_4139_MOESM1_ESM.png]

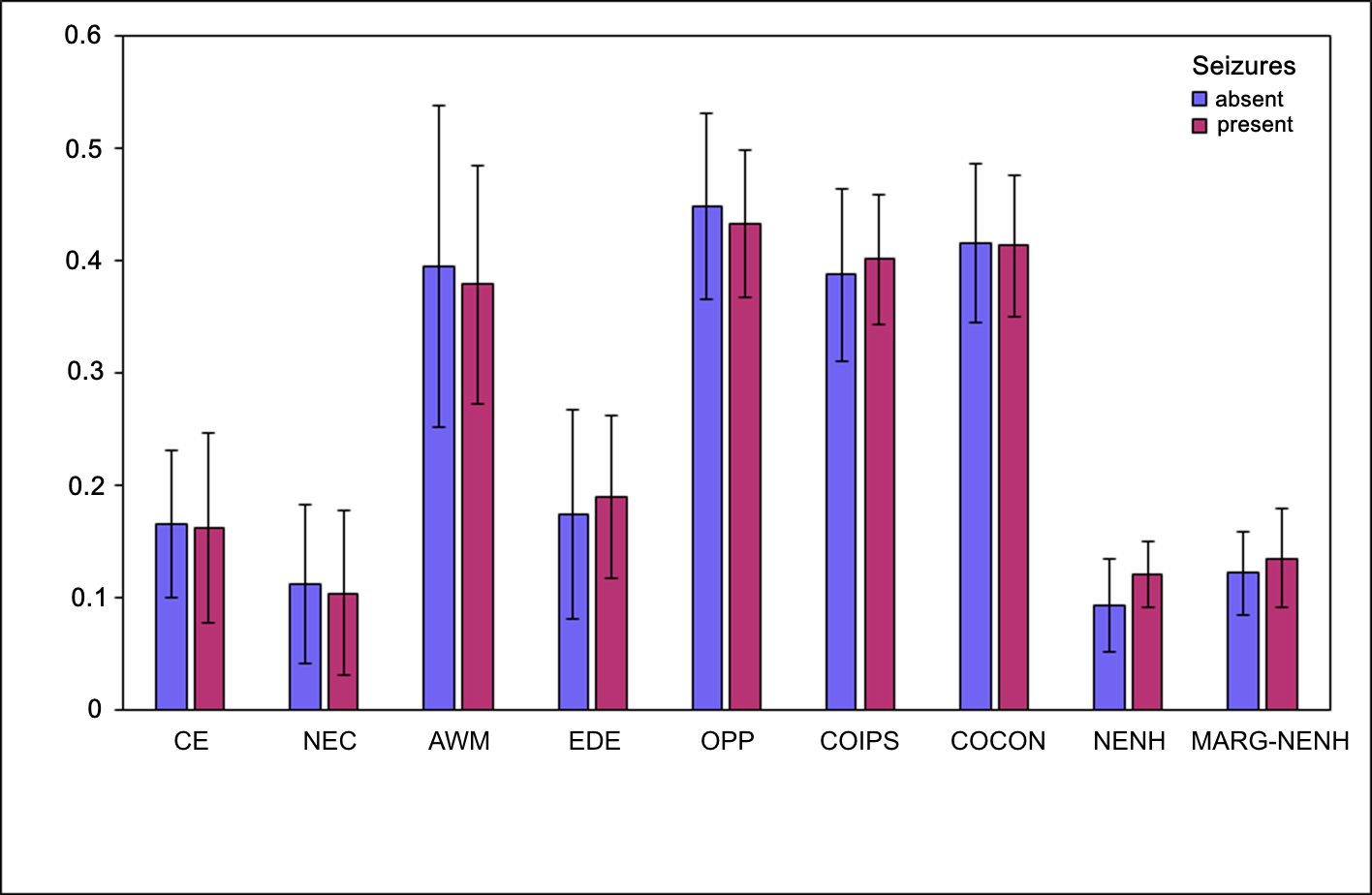

Supplement: Supplementary file 2 — The bar chart demonstrates the fractional anisotropy (mean ± SD) for different regions of interest as above defined (see Fig. 5) for cohort B. Data was dichotomized into patients with and without history of seizures [file 11060_2022_4139_MOESM2_ESM.png]
